# The Role of Play in the Social Development of Grey Seal (*Halichoerus grypus*) Pups with Comparative Notes on the Harbour Seal (*Phoca vitulina*)

**DOI:** 10.3390/ani14142086

**Published:** 2024-07-17

**Authors:** Susan C. Wilson

**Affiliations:** Tara Seal Research, 141 Victoria Road, Mablethorpe, Lincolnshire LN12 2AL, UK; suewilson@sealresearch.org; Tel.: +44-7742-451852

**Keywords:** grey seal, harbour seal, mother–pup, weaned pup, subadult, behavioural development, social play, affective touch

## Abstract

**Simple Summary:**

The social interactions of grey seal (*Halichoerus grypus*) pups at a salt marsh breeding site were observed to gain an understanding of the early development of sociality in this species. Pups interacted with their mothers immediately before and after suckling bouts, involving nose-to-nose and nose-to-body types of contact. However, some mother–pup pairs also engaged in play behaviours involving close body contact, with the mother usually taking the initiative by leaning her head over the pup, described as the play “invitation”. After the pups were weaned at ~2.5 weeks of age, they gathered around pools of water. There they interacted tentatively, often only touching whiskers or noses, or swam rapidly past a companion with much splashing. They attracted each other’s attention by adopting play-like displays with their flippers and heads extended, eventually following each other along channels connecting to the sea. Subadults reprised the contact play of mother–pup pairs; the components of this play are repeated during adult courtship and mating and thus personal relationships developed through nosing and contact play may underscore the social cohesion of the breeding colony. Harbour seals, *Phoca vitulina*, show the same behaviours, suggesting a similar social network prevailing in this, and possibly other, closely related species.

**Abstract:**

Juvenile grey seals are known to be highly social, interacting with contact behaviours interpreted as gentle play. However, minimal sociality of pups with their mothers and among weaned pups has been suggested. The present study aimed to observe the natural social interactions of pups to track the early ontogeny of their sociality. Pup behaviour at a salt marsh colony on the east coast of England was video-recorded. Pups interacted with their mothers around suckling bouts and after weaning as they gathered around pools. The records were transcribed to spreadsheets in 30 s time segments to estimate the frequency and co-occurrence of different behaviours. Mother-pup interaction comprised nosing contacts and sometimes contact play, involving one laying the head and fore-flipper over the other. Initial weaned pup encounters involved tentative nosing and defensive splashing, indicating contact shyness. However, socially orientated locomotor play, supine posturing, and exaggerated raising of fore- and hind-flippers led to reduced shyness and pups following one another towards the sea. Archive data on subadult interactions and on harbour seal behaviours were re-analysed. Gentle play-like contact between mother–pup, juvenile, and adult pairs is interpreted here as a universal mode of social bonding, underscoring the social structure of both grey and harbour seals.

## 1. Introduction

Understanding the ontogeny of social behaviour in any mammal species ideally requires observation of animals at different stages between dependent infancy to reproduction. Among placental mammals, there are two main dependent infancy stage types: altricial and precocial. Among the Carnivora, altricial infants are typical of terrestrial species, while infants of the aquatic pinnipeds are all precocial. However, the pinnipeds are again divided phylogenetically into sea lions and fur seals (Otariidae), and walruses (Odobenidae) and seals (Phocidae) [1]. 

The phocids diverged from the otariids around 25 MYA [2]. Sea lions and fur seals retained dependence on land for breeding, limbs sufficient for agile locomotion on land, and all breed on land; they are all polygynous and sexually dimorphic, with breeding males competing for mating privileges. Phocid evolution headed towards a more fully marine orientation with the development of a thick subcutaneous fat layer (“blubber”) and a more streamlined body shape with external limbs too reduced to assist locomotion on land [1,2]. The extent of land (or ice) dependence for breeding and the degree of polygyny varies widely between species, from relatively little (such as spotted seals and Caspian seals) to extreme (elephant seals). The otariids all have a maternal dependency period varying from about six months to a year, whereas all phocid species have a lactation period ranging from only a few days (hooded seals) to about six weeks (e.g., monk seals), followed by several years of independence as juveniles before becoming a potentially breeding adult [1]. 

During the later months of the lactation period, sea lion and fur seal mothers leave their pups onshore while they forage at sea; while their mothers are absent, pups gather in pods on the beach and interact socially, typically in a mode usually characterised as “play-fighting” [3,4,5]. Less is known about the social interactions of phocid seal pups, likely because of their more aquatic habitat. After weaning, elephant seal pups engage in “play fighting” on their natal beach [6,7], although play fighting has not so far been documented in other phocid pups. However, the elephant seal belongs to the southern monachine group, which diverged from the northern phocids ~20 MYA [2]. 

Grey and harbour seals are in a subfamily of northern phocids that probably diverged from other northern phocids ~6 MYA, while the grey seal (and its closest relative, the Caspian seal) diverged from the harbour seal ~2.5 MYA [8]. Grey seals (*Halichoerus grypus*) are sexually dimorphic, polygynous to a variable degree, and are dependent on ice or land for pupping. Adult grey seal social structure is complex, involving territoriality and dominance order among breeding adult males [9] and inter-year mate fidelity [10]. Harbour seals (*Phoca vitulina*) are only slightly sexually dimorphic and are less dependent on land for breeding [11]. Juvenile social interactions in both species can rarely be characterised as “play fighting”; rather, they are more appropriately described as “gentle play” [12] (pp. 308–310), in which the seals either on land or in the water engage in repeated or protracted gentle body contact in the water involving twisting or somersaulting over each other, termed *rolling* [11,13,14,15]. In the water, both species may engage in locomotor play, which includes splashing, bubble blowing, “torpedoing”, and “porpoising” or “high diving” [11,14,16]. 

The present article focuses on the social development of young grey seals as observed in their natural habitat. Some introduction to the history of the grey seal or its natural environment may therefore be helpful to understanding the behaviour of young seals. The grey seal was historically a typical ice-breeding phocid seal, with the edge of fast ice or drifting floes of pack ice being the optimum habitat [17,18], although since the end of the last Ice Age, most populations now breed on land. The average pup density on pack ice in the Baltic has been estimated at 0.2 pups/100 m^2^ compared to higher densities on land of up to 5.2 pups/100 m^2^ [18]. The female gives birth to only one infant, which interacts closely only with its mother. Lactation length averages ~18 d, at the end of which the mother leaves the pup abruptly. The weaned pups then stay onshore for ~1–4 weeks (average of ~3 weeks) before departing for the sea [19,20,21]. During this period, pups at the relatively dense land-based colonies may encounter one another, whereas it seems likely that pups more sparsely distributed on ice floes rarely have that opportunity. 

After departure for the sea, the pups may cover considerable distances over the next few months, exploring, gaining experience in navigation, and learning to forage [22]. After leaving their natal site, therefore, pups may have little social contact with conspecifics for some weeks or months. This likely means that the lactation and post-weaning periods are critical for pup primary socialisation [23]. 

Social contact play between mother and pup has been documented in harbour seals [15,24,25] but the record for grey seals is less clear. A study of the social development of a zoo-born female pup (“Kara”) described brief contact play bouts immediately before or after suckling, during which mother and pup leaned their heads over each other and made gentle nose-to-nose contacts [23], which have been previously described for juveniles during field observations [14]. However, in a field study, observed pup play was limited to wriggling movements and did not involve interaction with the mother [19]. Pup-to-mother nosing contacts only occurred <1% of the time in early lactation and up to ~2% of the time in mid or late lactation [19,26]. Pup-to-mother body nosing was considered to relate to pup soliciting suckling [19] and play-like interaction to be the mother inciting suckling [27]. 

After weaning, the grey seal pup has been described as *unsociable and hostile to all other seals* [28] or as aggressive towards other pups [29]. The zoo study of pup “Kara” post-weaning found her to be initially *shy of any social contact, even with her mother*, responding defensively by splashing or snorting. However, two months post-weaning, Kara began to follow the other seals, often nosing their hind-flippers and with locomotor play, although close contact was not observed [23]. Post-weaning pups on the Isle of May (Scotland) gravitated to inland pools where they started to play exuberantly, with splashing and somersaulting. However, the play then observed was self- or object-directed; pups occasionally approached one another, but further contact was not seen [19]. 

Thus, the social experience of young grey seal pups appears to be very limited, although subadults are known to be highly social at the water’s edge of haul-out sites [14,30]. Social interaction usually takes the form of one seal leaning its head over the other’s back as an “invitation”; play interaction then proceeds when this *head-over* contact is reciprocated and continues with repeated reciprocal *head-over* and variations thereof [14]. The play appears to be socially contagious, with several dyads sometimes playing simultaneously [30]. 

The aim of the present study is to delve further, by field observation, into the grey seal pups’ early experience of positive social interaction before and after weaning, their social interaction as subadults, and speculate how this may underlie the social cohesion of breeding colonies and courtship by breeding males. Because mother–pup social interaction has previously been found to occur infrequently, and mainly in relation to suckling bouts, this study aimed to focus on mother–pup social interaction around suckling bouts. Finally, the commonality of grey and harbour seal pup and subadult play is considered in terms of their social structure, respective habitats, and phylogenetic relationship. 

## 2. Animals and Methods

### 2.1. Observation Site

**Figure 1:**
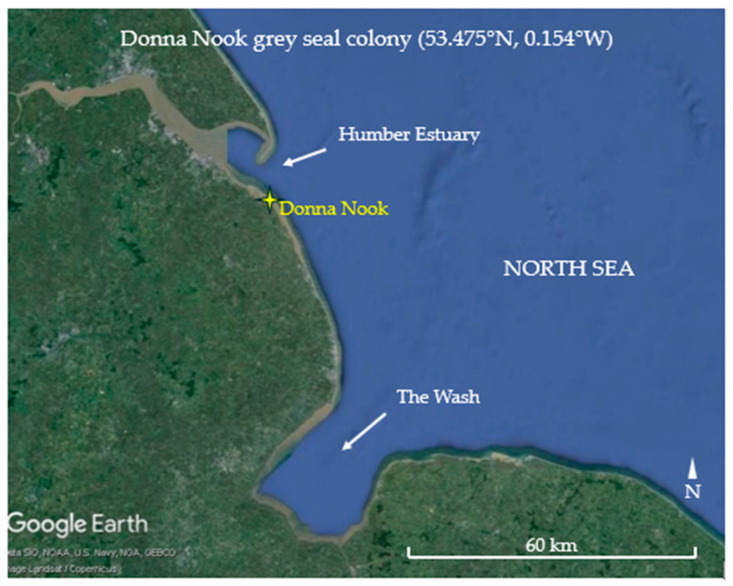
Location of grey seal breeding colony at Donna Nook (map: Google Earth).

Grey seal mother–pup pairs and weaned pups were observed at Donna Nook, north-east England, UK (Figure 1), during the November–December pupping seasons in 2022 and 2023. 

The grey seal pupping site at Donna Nook (DN) is a salt marsh extending inland for about 0.75 km from the sea; the areas used by the seals for pupping consist of grassy areas interlaced with pools and saltwater channels. Grey seals haul out on the coastal beach throughout the year, but the pupping ground is almost exclusively inland on the salt marsh, where the pups are safe except during extremely high tidal surges. A total of 2207 and 2205 pups were recorded in 2022 and 2023, respectively (https://www.lincstrust.org.uk/get-involved/top-reserves/donna-nook/weekly-update; accessed 30 January 2024). The pup density equates to that typical of inland sites [18]. Births occur from the end of October to the 4th week in December, with peak pup numbers at the beginning of December; preweaning mortality in 2023 was estimated at 6.8% (M. Blissett, R. Taylor, pers. comm., 22 January 2023). In 2023, the maximum number of adult males was estimated at 500 on December 01, when 1559 adult females were counted (M. Blissett, pers. comm., 31 December 2023), suggesting a sex ratio in the salt marsh breeding area of approximately 1♂:3♀. 

### 2.2. Behaviour Recording 

Mother-pup (MP) pairs and weaned pups (WPs) at DN were observed and filmed by the author from behind a fence separating the human public from the seal pupping areas. The behaviour of MP pairs was recorded on 16 dates in 2022 between 24 November and 17 December and on 20 dates in 2023 between 3 November and 16 December. The behaviour of WPs was recorded on five dates between 9 December 22 and 11 January 23 and eight dates between 9 December 23 and 31 December 23. Seal behaviour was video-recorded using a Panasonic camcorder (up to 70× zoom) and a Nikon Coolpix (up to 24× zoom) in 2022, and a Panasonic Lumix (up to 60× zoom) in 2023. 

Because suckling is known to occur only about once every six hours, or ≤7% of the time [19,26,27], the interaction between focal MP pairs was recorded on an opportunistic basis by scanning the breeding colony for MP pairs engaged in pre-suckling or post-suckling or other social interaction. 

The recorded MP behaviour was classed by the author into four sequence types: *pre-suckle*, *inter-suckle* (i.e., the interval between a pup stopping suckling and starting again), *post-suckle,* and *unrelated* (i.e., when suckling was not observed either before or after the activity). A single MP record includes one or more sequence types (Table 1). The observations were further classified into five observable stages of pup physical development [31], i.e., stage 1—newborn, thin and clumsy movement, birth fluid-stained lanugo fur, wet umbilicus; stage 2—white lanugo coat, pup “tubular” in shape; stage 3—white lanugo coat, pup very fat; stage 4—moulting lanugo over body; and stage 5—fully moulted. Newborn pups within the first 90 min of birth or up to their first suckling were classed separately as stage 1nb (Table 1). Because there were relatively few MP records with stage 4 or 5 pups, these records are combined for the analysis.

As the season progressed and mothers began to depart for the sea, WPs collected into groups, some resting on grass or sand and some active, mainly around pools of water. Behaviour was recorded of groups of WPs seen to be active in the vicinity of water.

WPs, both ♂ and ♀, at stages 4 or 5 were distinguishable by their pelage pattern (males generally being darker than females and without a clear demarcation of the dorsal and ventral surfaces, as in females). The WP behaviour sequence recording began on an opportunistic basis when a pup was seen to be active in the vicinity of one or more other pups. Recording of a sequence ended when the pups were no longer visible or ceased being active 

### 2.3. Video Recording Transcription and Analysis

The behaviour of a total of 114 different grey seal MP pairs at Donna Nook was recorded in the 2022–2023 seasons. For MP behaviour, the number of nose-to-nose and nose-to-body contacts (to each body region) was recorded for each *pre-suckle*, *inter-suckle*, *post-suckle,* and *unrelated* interaction bouts. Each nosing contact recorded received a score of 1, although prolonged single nosing contacts lasting more than ~1 s received a score of 2. MP contact play was operationally defined as a sequence in which either the M or P leaned its head or chin over the other (*head-over*). Other behaviours recorded included the adoption of the *supine* position, *open-mouth* “play face”, *play movement* (lateral body movement or exaggerated galumphing), fore-flipper over (*ffl-over*) contact, and the M scratching the P towards the nipples (*scr*). The MP video records were transcribed by the author and recorded in Excel spreadsheets available in the Supplementary Materials. These materials include a catalogue of all MP pairs recorded (Table S1), the behavioural record in 30 s segments for each MP (Table S2), a record of all body nosing scores to each body region (Table S3), and a record of all body nosing scores for play vs. non-play sequences (Table S4). 

The behaviour of WP pairs during 22 social interaction sequences (November–January 2022/23), a further 84 sequences (November–December 2023), and records of single weaned pups apparently active alone were transcribed and recorded in Excel spreadsheets (Table S5). 

Abbreviations commonly used in all the Excel records in this study (Tables S1–S5 and Tables S7–S9) are listed in Table S6. 

### 2.4. Grey Seal Subadult Play 

Previously unpublished archive data from observations by the author at the “Red Wilderness” [32] site in west Wales, UK (approx. 52.0208° N, 4.858° W), recorded in the spring of 1986 and 1987, were revisited. Seals had been observed from a cliff above the haul-out site. A series of still photos of juvenile and subadult (referred to as “subadult” (SA)) dyadic play were recorded using a 500 mm lens. 

A different playing dyad was selected for each sequence on any given day, resulting in a total of 21 sequences recorded (11♂♂, 9♂♀, and 1♀♀). Within each sequence, a new frame was taken each time the dyad changed position. 

For the SA ♂♀ sequences, the amount of swapping of the *head-over* role between the partners was noted; swapping of the *head-over* role in MP play was also noted and compared with SAs ♂♀ (Table S7). 

Overlapping body contact was estimated by tracing each frame of the photographed dyad onto transparent paper, and then estimating, using graph squares, the amount of overlapping body contact area as a percentage of the average 2-dimensional area of both seals of the dyad.

One mating sequence between a breeding male and female at a pupping beach (Pwllderi, St David’s, West Wales, UK) was photographed from an overhanging cliff and overlapping body contact was estimated in the same way as for subadult play. 

Play by harbour seal (*P.v. concolor*) MP pairs and subadult (SA) dyads at Sable Island (SI), Nova Scotia (44.086° N, 59.885° W), was observed by the author between 15 May and 19 June 1973 [15]. 

The SI harbour seal population is isolated by ~200 miles from the harbour seals of the mainland coast of north-east America. Much social interaction by MP pairs and SA dyads takes place onshore at the water’s edge, whereas in other populations it mostly takes place in the water [11,13,14,16,24,25,33]. 

The behaviour was observed through binoculars from a vantage point on the sand dunes and a voiced narrative account of the behaviour was tape-recorded and later transcribed, incorporating a time check at 30 s intervals. This narrative for 10 sequences involving mother–pup pairs, and a sample of sequences for subadult dyads, is re-evaluated here by transferring the previously transcribed narratives into Excel spreadsheets (Tables S8 and S9) according to the method (above) for transcribing the 2022–2023 grey seal mother–pup video recordings.

## 3. Results

### 3.1. Grey Seal Mother-Pup (MP) Pairs

Of the 167 interaction sequences recorded (2022 and 2023 seasons) of pups after the day of birth, only 16 were observed outside the context of a suckling bout (Table 1). For the rest of the time, the pups lay asleep a few metres away from their mothers. 

MP pairs usually exchanged nosing contacts before and after suckling, and sometimes during intervals in a suckling bout. A nosing exchange led to a play bout when either the M or P leaned the head over the other, referred to as *head-over* contact. However, this behaviour was only observed with 26 of the 108 MP pairs with pups past the newborn (nb) stage. Play was sometimes only momentary (lasting ≤ 1 min). The remaining play sequences ranged from 90 s to 10.5 min, while seven bouts in water lasted a relatively long time (average 4.0 min). 

The play occurred with the highest frequency during *unrelated* sequences and least often during *pre-suckle* interactions. The stage 4/5 pups had the highest frequency of play (Table 1) and of these, six pups were ♀ and three were ♂. 

**Figure 2:**
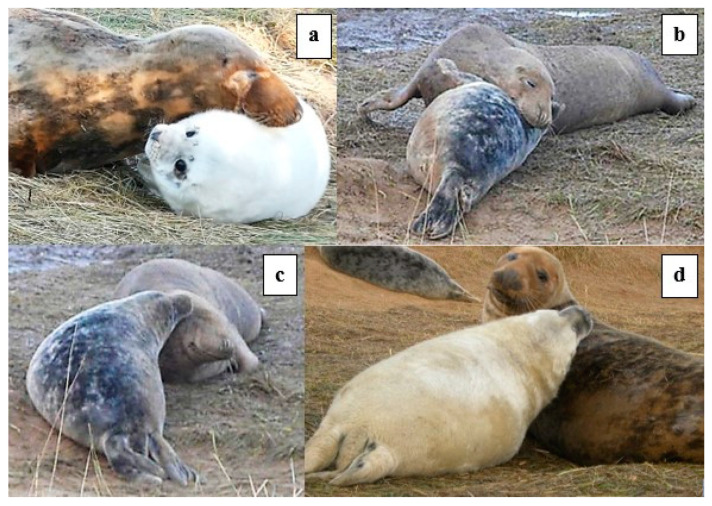
Examples of *head-over* play “invitation”. (**a**,**b**) M to P; (**c**,**d**) P to M.

Play behaviours included foremost the defining *head-over* contact (Figure 2). However, only 17 of 43 play bouts included at least one swap of the *head-over* role, and in these 17 bouts, the overall M:P ratio of head-over contact was 2.4:1, i.e., the mother was most often the prime mover of interactive play. Of the 34 bouts where there was no *head-over* contact reciprocation, 20 bouts were initiated by the M and 14 by the P. One stage 3 ♂P was seen to make head-over contact 16 times with no reciprocation by the M (Figure 2d). However, even when the *head-over* contact was not reciprocated, the M or P often responded by lying *supine*, fore-flipper over (*ffl-over*) touching, or with the *open-mouth* play face. 

The occurrence of behaviours specifically associated with suckling was relatively infrequent during play (P vocalising (3% of 149 30 s segments), M *scr* (26%), and P nosing the nipple region (3%)).

**Figure 3:**
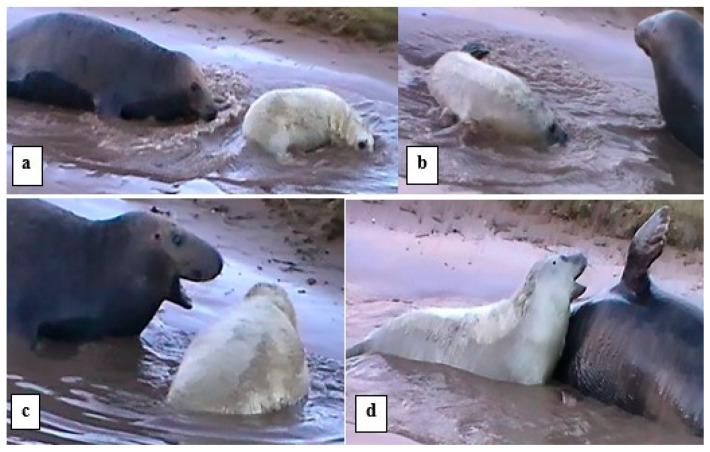
Examples of *play movement* in water and *open-mouth* “play face”. (**a**) *Play movement* in the water by the M and P; (**b**) P continues *play movement*; (**c**) *open-mouth* gesture by M; (**d**) *open-mouth* gesture by the P; M making raised *ffl play-display*.

Figure 3 shows examples of play movement in the water and the *open-mouth* “play face”. 

**Figure 4:**
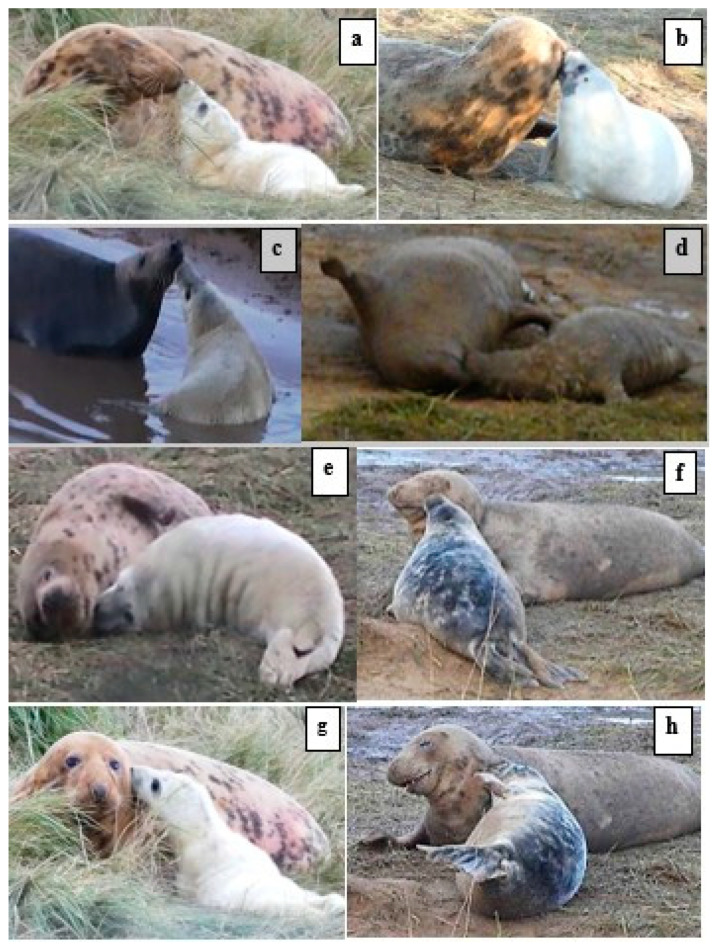
Examples of MP *nose-to-nose and* unilateral P-to-M nose-to-body (n) contacts during play interaction: (**a**) nose-to-nose; (**b**) P nuzzles M’s muzzle; (**c**) P n M’s mouth; (**d**) P n M’s mouth while M is *supine* (M and P covered in wet mud); (**e**) P n M’s throat; (**f**) P n M’s side-of-face; (**g**) P n M’s side-of-neck (**h**); P n M’s side-of-neck; and (**b**,**h**) M responds with relaxed *open-mouth*.

**Figure 5:**
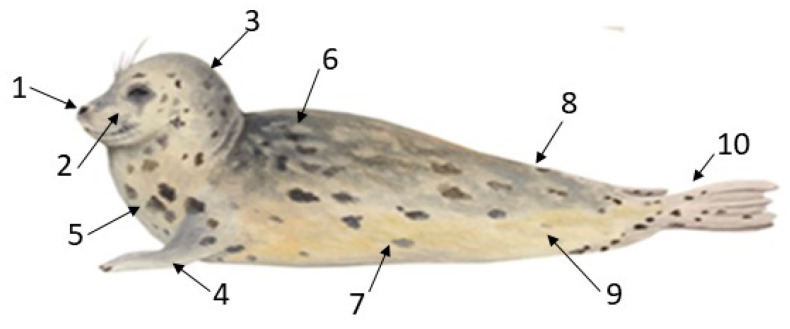
Mother body regions nosed by pup: 1 = nose; 2 = muzzle, face; 3 = top/back of head/side of neck; 4 = fore-flipper; 5 = throat/chest; 6 = shoulder/back; 7 = mid-venter/flank; 8 = rump; 9 = rear venter; and 10 = hind-quarters/hind-flippers.

Nose-to-body contacts by the pup to the mother during play were directed mainly towards the mother’s face and head and neck regions (Figure 4). Mutual nose-to-nose contact (Figure 3a; mother’s body region 1) was the most frequent (24%) of the pup’s total nosing contacts (Figure 5; Table 2). Unilateral nosing contacts by the pup to the mother’s muzzle, head, neck, and chest (body regions 2–5) accounted for a further 54% of the pup’s total nosing score (Figure 5; Table 2), while the mother’s shoulder, back, rump, abdomen, and hind-flippers (body regions 6–10) were nosed least often (total 22%; Figure 5; Table 2).

**Figure 6:**
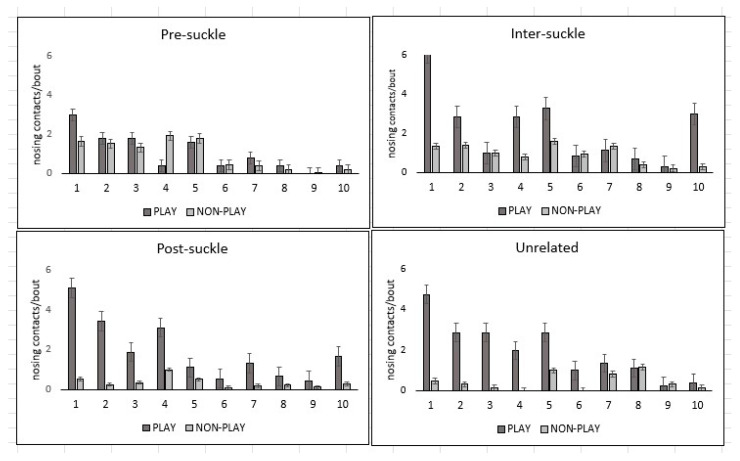
Comparison between the number of pup-to-mother (PnM) nosing contact scores to each of the mother’s body regions 1–10 (Figure 5) per play or non-play interaction bout. No. of bouts of each type are shown in Table 1.

Mutual nose-to-nose contact and the P nosing the M occurred more frequently in play vs. non-play bouts during *inter-suckle*, *post-suckle*, and *unrelated* bouts, although this was not apparent during the *pre-suckle bouts* (Figure 6). 

**Figure 7:**
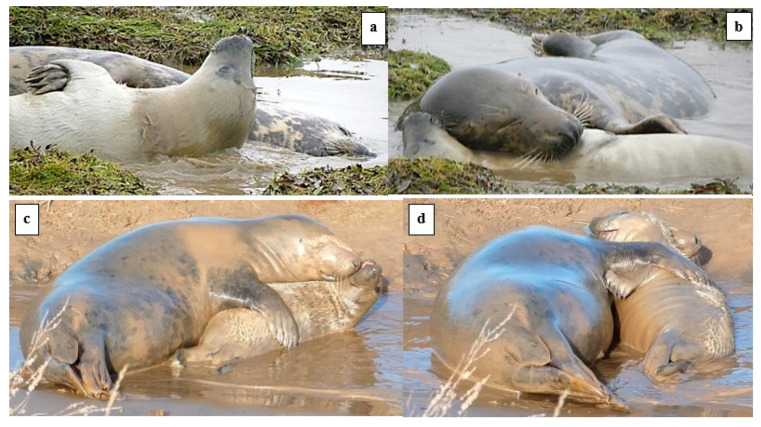
Static close contact during play with closed eyes: (**a**) mutual *ffl-over* contact, (**b**) mutual *head-over* and M *ffl-over* contact, (**c**) M *head-over* and *ffl-over* contact (P *open-mouth*), and (**d**) P *head-over* and M *ffl-over* contact.

Play sometimes led the pair into almost static contact, with notably relaxed body tone, fore-flipper over (*ffl-over*) contact, and closed eyes (Figure 7).

**Figure 8:**
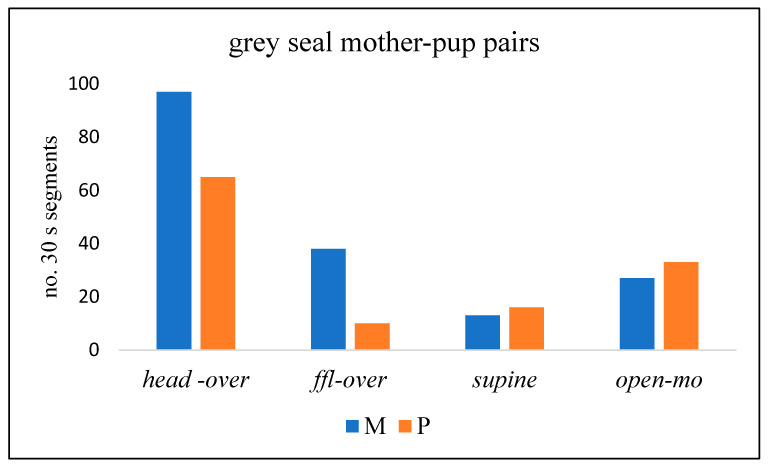
No. of 30 s time segments of play of grey seal mother-pup (MP) pairs in which four key behaviour components occurred (Donna Nook, 2022–2023).

Comparison of the contributions of the M and P in four key components of play (Figure 8) indicates that M made *head-over* and *ffl-over* contact more often than the P, whereas the P adopted the *supine* position and *open-mouth* at least as often as the mother (*p* = 0.002; chi-squared test). 

### 3.2. Grey Seal Weaned Pups (WPs)

Immediately after weaning at Donna Nook, the pups congregate in groups on the marsh grass, usually with about one pup’s body length between individuals. Socially orientated behaviour of WPs was recorded in the water slightly more often than on the grass (265 of a total 449, 30 s). Behaviours transcribed from the video recordings are listed in order of prevalence (Table 3). 

**Figure 9:**
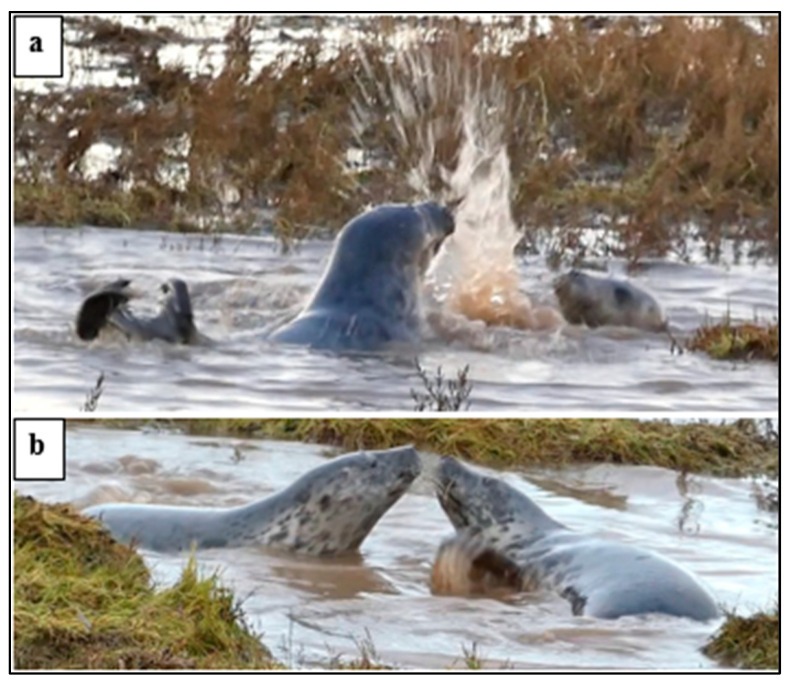
*Defensive* and tentative approaches by weaned pups (WPs): (**a**) *defensive* splashing; (**b**) *whiskers*.

The most frequent social behaviours of weaned pups were approach, follow, and looking at another pup (Table 3). Behaviours interpreted as play (*splash*, *play movement*, *play display*, and lying *supine*) occurred in 13–24% of 30 s segments, although the *open-mouth* and *head-over* behaviours occurred rarely. *Defensive* splashing and tentative *whiskers* (Figure 9) were relatively infrequent (Table 3). 

**Figure 10:**
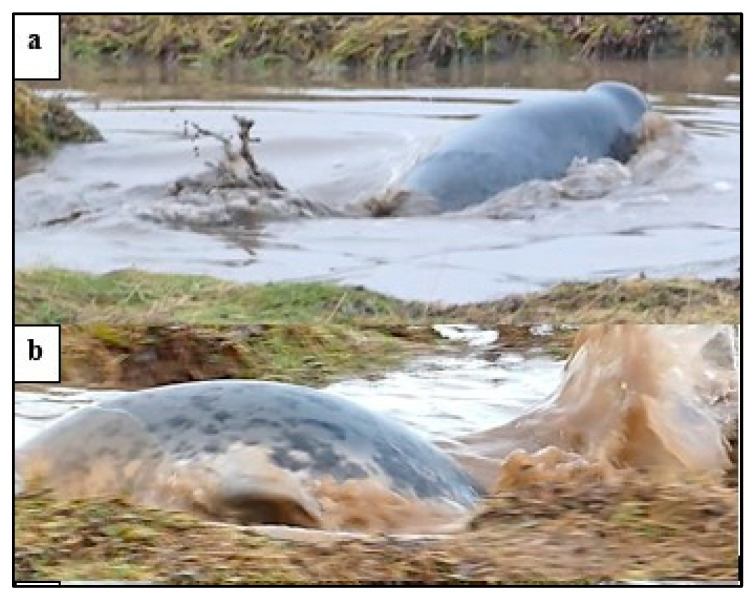
Play *splash* and *play movement* by weaned pups (WPs) in the water: (**a**) rapid *play movement* with body and hind-flipper *splash*; (**b**) play high dive with a *splash*.

*Play splash* (Figure 10) accounted for 107 of the 265 30 s time segments recorded in the water. 

**Figure 11:**
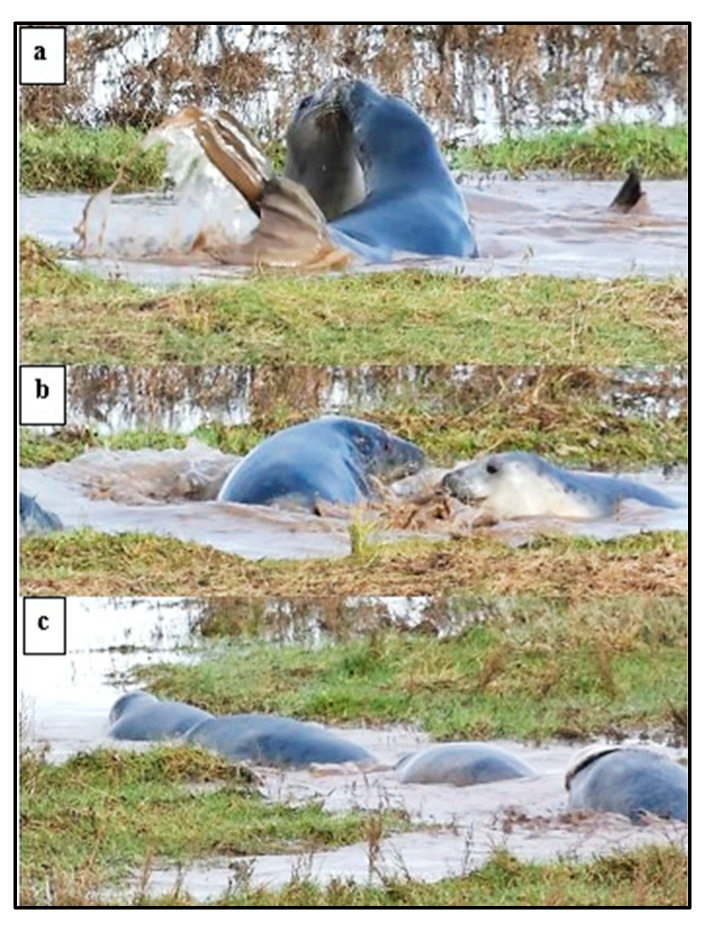
An example of *follow* by weaned pups (WPs) in the salt marsh channel, preceded by social interaction: (**a**) two pups *nose-to-nose* with mouth contact combined with hind-flipper raised in *play-display*; (**b**) mutual *look-at* and play *splash*; and (**c**) four pups *follow* along the channel.

*Follow* occurred across the salt marsh grass or water channels and was observed with up to four pups following in a line (Figure 11).

**Figure 12:**
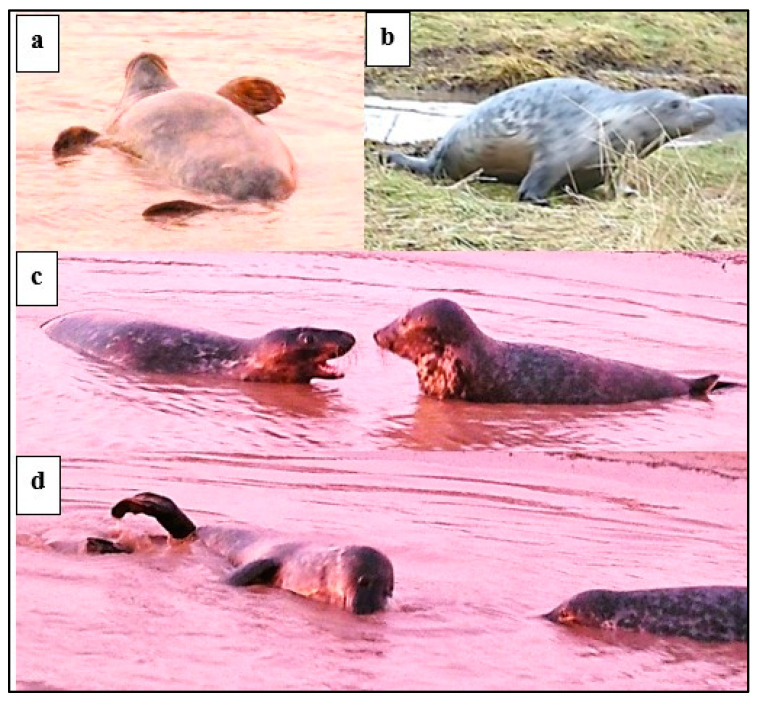
Examples of positions adopted in a social context by weaned pups indicative of a play “attitude”: (**a**) *supine*, (**b**) “galumphing” *play movement* on shore; (**c**) *approach* with *open-mouth*; and (**d**) *approach* with direct *look-at*, relaxed body tone, and hind-flipper waving.

Some positions adopted by the pups, almost always in a social context, were considered to indicate play “attitude”, including lying *supine* with a relaxed body tone and *open-mouth* play face (Figure 12). 

**Figure 13:**
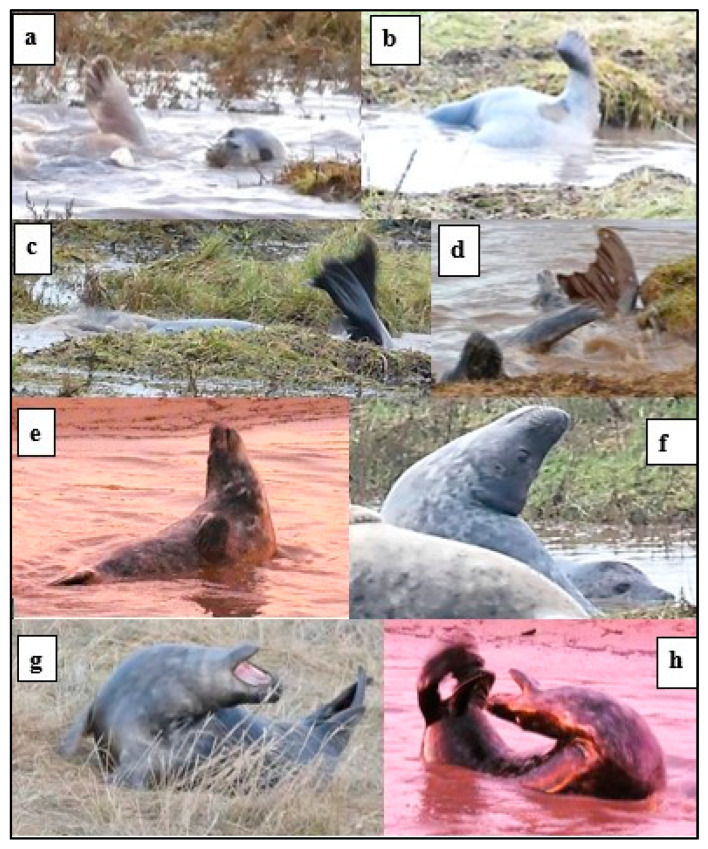
*Play-display* by weaned pups (WPs): (**a**,**b**) fore-flipper raised (*ffl*); (**c**,**d**) hind-flipper raised (*ffl*); (**e**) head stretch up (*H*); (**f**) head stretched back (*H*); and (**g**,**h**) head stretched right back (*H*) with raised hind-flipper (*hfl*) and *open-mouth*.

**Figure 14:**
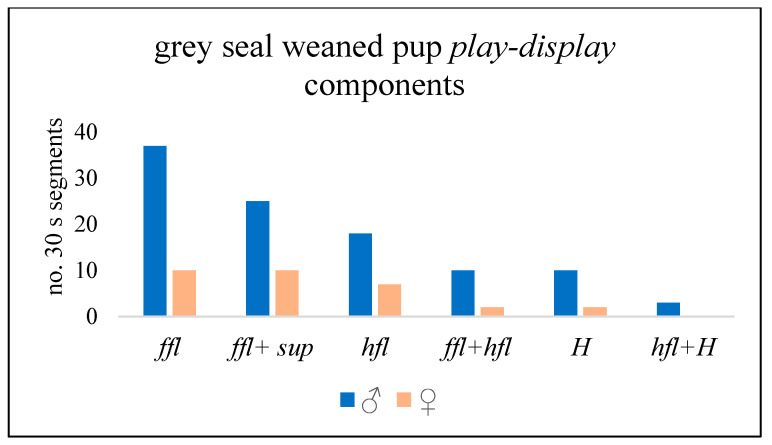
No. of 30 s time segments in which key components of grey seal weaned pup (WP) *play-display behaviour* occurred. *ffl* = fore-flipper raised; *sup* = supine; *hfl* = hind-flippers raised; and *H* = head raised or stretched backwards.

*Play-display* (Figure 13) was more often recorded by ♂ pups (49/65 records) than by ♀s, and 52 of the 65 recorded occurrences were in the water. Raising one or both fore-flippers (*ffl*) was the most frequent display, followed by *ffl* together with *supine*. Hind-flipper raising (*hfl*) and stretching the head up or backwards (*H*) were the next most frequent displays, while *hfl* plus *H* occurred least frequently (Figure 14). Displays by ♀ pups were most often *ffl* or *hfl*, or *ffl* plus *sup* (Figure 13 and Figure 14), whereas *H* and *H + ffl* seemed to occur most often by ♂ pups; one ♂♂ pair displayed to each other over a period of ~18 min (Figure 12 and Figure 13), but no physical contact was recorded.

A contingency table of the occurrence of the WP behaviours indicated that *splash* and *supine* were most likely to occur in the same 30 s as *play-display*. However, *splash* and *play movements* were less likely to occur in the same 30 s as *follow*, while *supine* did not co-occur with *follow* at all (*p* < 0.0001; chi-squared test; Table 4). 

### 3.3. Grey Seal Subadult (SA) Play 

**Figure 15:**
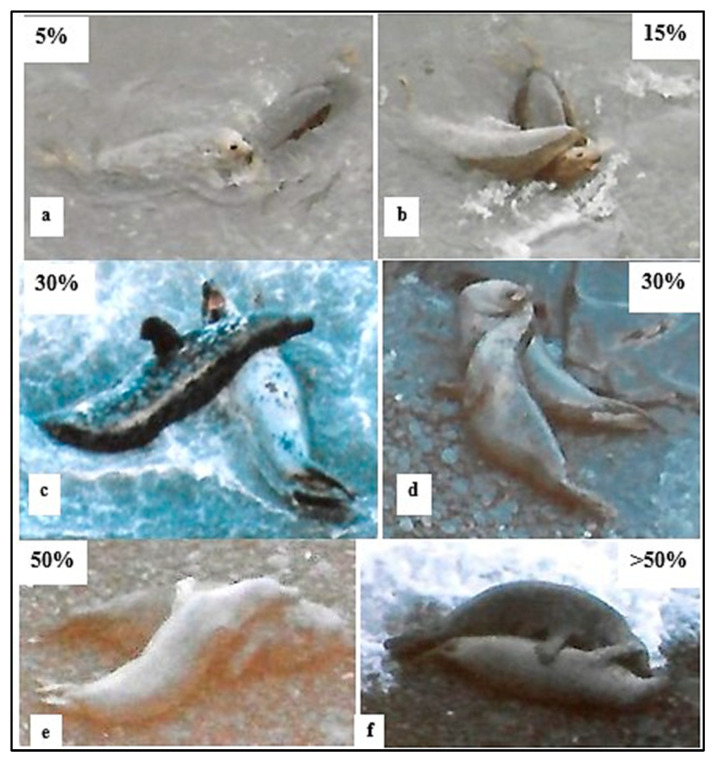
Examples of variable amounts of mutual body contact during play of subadult (SA) grey seals (Red Wilderness, West Wales, 1986–1987). Approximate amounts of % of body contact indicated (see text); note *open-mouth* “play face” in (**c**,**d**) and raised fore-flipper (*ffl*) in (**c**); (**a**,**b**,**e**,**f**) are ♂♀ dyads; (**c**) is ♂♂; and (**d**) is ♀♀.

Interactions witnessed between subadult seals occurred at the water’s edge and were playful in nature, with a relaxed body tone and often including an *open-mouth* “play face”. Play was always dyadic, with each seal leaning its chin, head, or fore-quarters over its partner, or lying side-by-side or venter-to-venter (Figure 15). Young grey seals also continue contact play in three dimensions in the water (Figure A4), although poor visibility precluded this from being photographed in the 1986–1987 study.

The core play contact pattern of subadult dyads was *head-over*, i.e., one seal leaning the head over the back of the other’s neck (Figure 15). Contact variations included leaning most of the body over the other; one seal lying across the other’s throat while it is *supine*; laying one or both fore-flippers on the partner, chest-to-chest; and “facing backwards”, placing the hind-quarters over the partner. One partner occasionally pushed its head under the other’s chin (to “create” *head-over* contact by the other) or the partners entwined their necks together in a mutual *head-over* position. 

The estimated amount of overlapping body contact between the playing SA dyad varied widely within and between different play bouts, ranging from nose-to-body contact only through >50% of the body surface in contact: 5% contact was approximately equivalent to one seal’s chin over the other; 15% contact was chin-and-throat-over; 30% was chin-throat-and-chest-over; 50% included the mid-venter; and >50% occurred when one seal was lying on top of or lying in close contact with its partner (Figure 15). Approximately 15% was the most frequent body contact level observed for all dyads (Figure A5). The ♂♂ dyads had more 5% contact and less >30% contact than ♂♀ dyads (*p* = 0.000; chi-squared test), although there was no discernible difference in contact levels between the ♂♀ dyads and the single ♀♀ dyad. 

**Figure 16:**
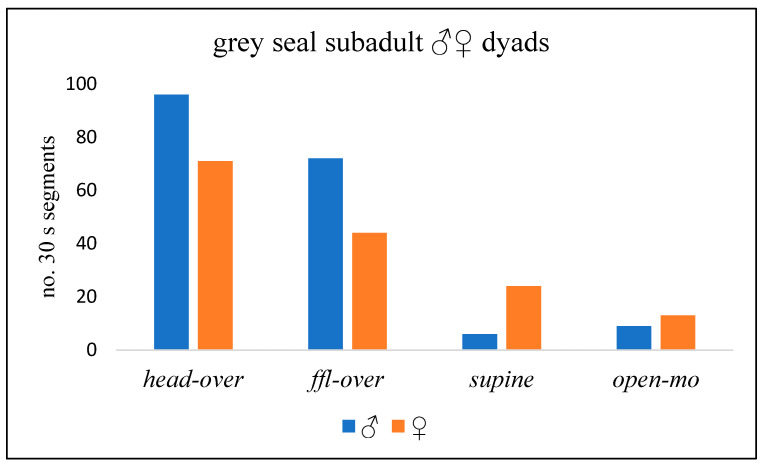
No. 30 s time segments of grey seal subadult (SA) ♂♀ dyadic play in which four key behaviour components occurred (Red Wilderness, West Wales, 1986–1987).

In eight ♂♀ play bouts, the ♂s engaged in more *head-over* and *ffl*-*over* behaviour than the ♀s overall (Table A1), while the ♀s generally adopted the *supine* and *open-mouth* play face slightly more often (Figure 16; *p* = 0.000; chi-squared test). In all play bouts recorded, the partners swapped the *head-over* position at least once (Table A1), but there was only a weak relationship between the number of swaps during a play bout and its duration (Spearman coefficient = 0.22). 

The play patterns of the SA dyads and the MP pairs were qualitatively similar, although some quantitative differences were detected: in ♂♀ dyads, the ♂s engaged in relatively more *ffl-over* behaviour than the mothers but less *open-mouth* (*p* < 0.0001), while the mothers engaged in relatively more *head-over* and more *open-mouth* behaviour than the subadult ♀s (*p* = 0.011) (Table 5). 

Grey seal courtship and coitus involve the ♂ leaning his head, parts of his body, and fore-flipper over the ♀ (Figure A5). The body contact amounts measured for one mating pair suggest a greater amount of overlapping contact >15% than for subadult play interactions (Figure A6). 

### 3.4. Harbour Seals

#### 3.4.1. Harbour Seal Mother-Pup (MP) Pairs

**Figure 17:**
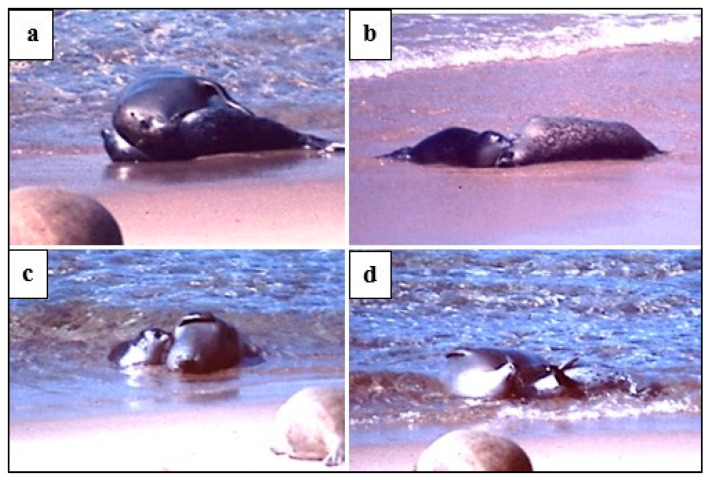
Play between harbour seal mother-pup (MP) pairs at Sable Island (May–June, 1973). (**a**) M lays head, chest (*head-over*), and fore-flipper over (*ffl-over*) P; (**b**) P lays chin over M’s throat, M semi-supine; (**c**) P noses M’s shoulder, M *supine*; and (**d**) play “attitude”—M leans back to P so their heads are in contact, M *semi-supine*, P *supine*, waving fore-flippers (*ffl*).

**Figure 18:**
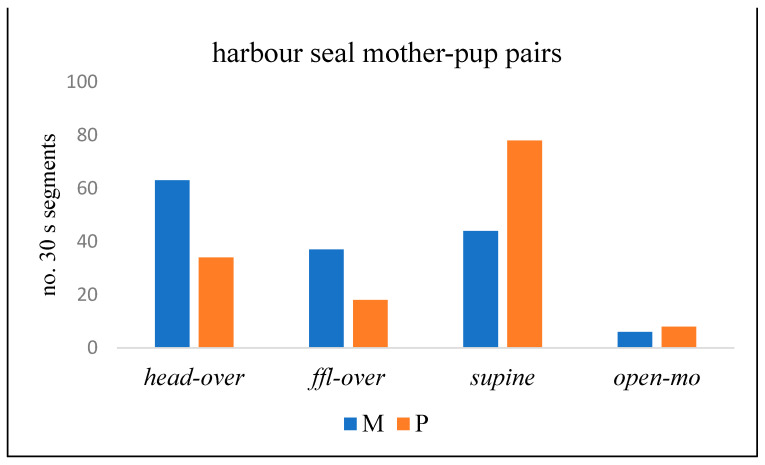
No. of 30 s time segments of play of harbour seal mother-pup (MP) pairs in which four key behaviour components occurred (Sable Island, May–June 1973).

Play by harbour seal MP pairs on Sable Island was observed in the surf at the water’s edge and as the pairs swam together in and out of the water (Figure 17). Key play behaviours were, as for grey seals, *head-over*, *ffl-over*, *supine,* and *open-mouth* play face. Ms exhibited *head-over* and *ffl-over* behaviour relatively more often than Ps, while Ps adopted the *supine* position more often than Ms (Figure 18; *p* < 0.0001; chi-squared test). 

In the water, Ps engaged in play splashing and locomotor *play movements* including “whooshing”, rearing up or high diving forwards or backwards, “streaking” at the surface, and somersaulting. 

#### 3.4.2. Harbour Seal Subadult (SA) Play

**Figure 19:**
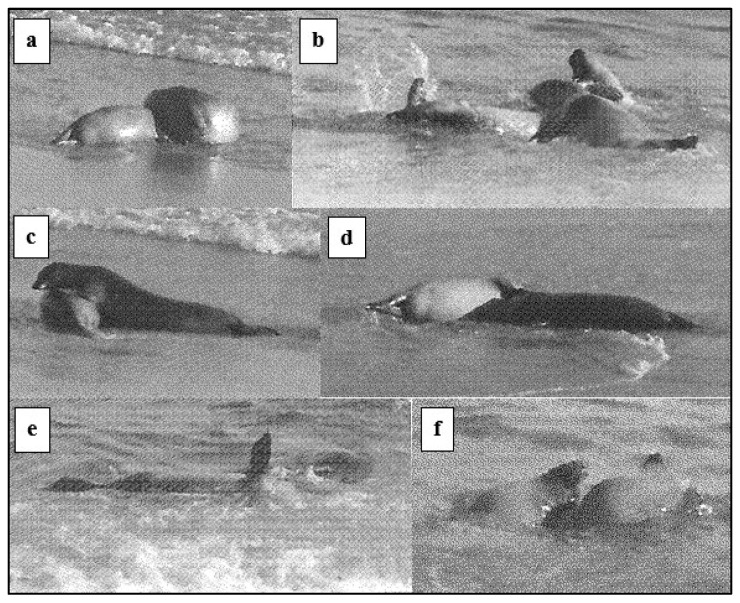
Play positions of harbour seal subadult (SA) dyads, Sable Island, May–June 1973: (**a**) *head-over*; (**b**) *head-over* with 2nd seal *supine*; (**c**) *head-over* with *ffl-over*; (**d**) *ffl-over*; (**e**) *ffl*; and (**f**) *rolling* head-to-head.

**Figure 20:**
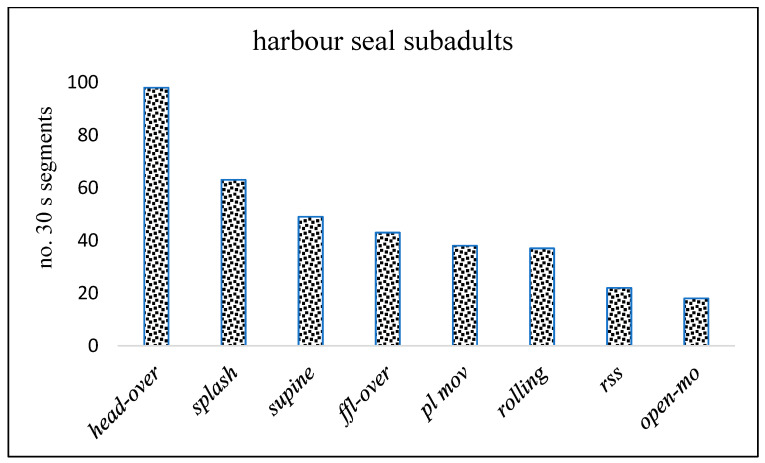
No. of 30 s time segments in which key components of harbour seal subadult (SA) dyadic play behaviour occurred. (Sable Island, May–June 1973).

Harbour seal SA dyadic play occurred at the water’s edge and in the tidal shallows (Figure 19 and Figure 20). The prominent behaviour was *head-over* (Figure 19a–c), often accompanied by *supine* (Figure 19b), *ffl-over* (Figure 19d), and *open-mouth. Rolling* involved the dyad twisting or wheeling around each other in continuous contact. Head-to-hind-flipper *rolling* was usually accompanied by splashing, while head-to-head *rolling* (Figure 19f) sometimes became slower with the dyad briefly in a static contact position in close contact, sometimes venter-to-venter, with fore-flippers around each other. 

Harbour seal SA *play movements* (noted in Figure 20) included “streaking” just below the surface, high diving, hind-quarter lateral movement, and sometimes synchronous “log-rolling” in parallel on the sand. Raised or waving fore-flipper (*ffl*) (Figure 19e) was recorded on 11 occasions. “Rifle-shot” splashing (*rss*) sometimes occurred by one of a dyad and was also executed by single SAs in the water near the focal dyad (noted in Figure 20). 

#### 3.4.3. Comparison of Harbour and Grey Seal Play Patterns

Harbour seal MP pairs displayed relatively more *ffl-over* and *supine* but less *open-mouth* behaviour than grey seal MP pairs (Table 6a; *p* < 0.0001; chi-squared test). Harbour seal SA dyads displayed relatively slightly more *supine* and less *ffl-over* behaviour than the grey seal SAs, although the difference was not significant ((Table 6b) *p* = 0.125, chi-squared test). 

## 4. Discussion

### 4.1. Grey Seal Mother-Pup (MP) Pairs

**Figure A1:**
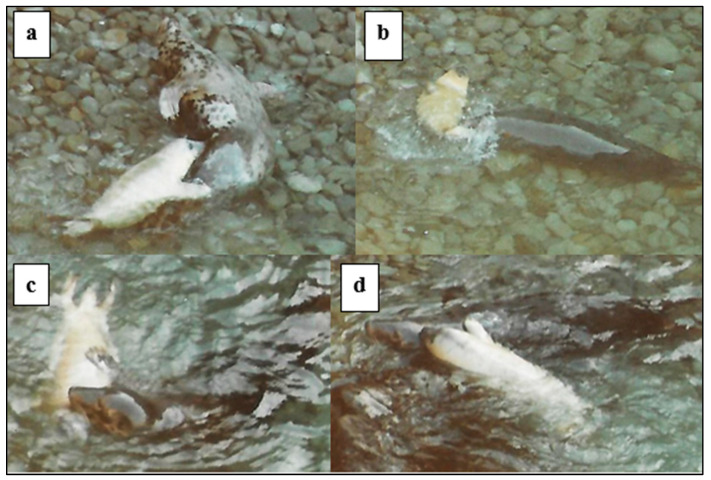
Aquatic play between a grey seal mother and pup at a coastal site (Pwllderi) in West Wales: (**a**) mother is *supine*, pup noses her rear flank, its fore-flipper over (*ffl-over*) her hind-quarters; (**b**) pup is *supine*, making hind-flipper splashes, mother noses its rear flank; (**c**) mother leans head over pup’s back-of-head (*head-over*) and makes *ffl-over* contact with the pup’s back; and (**d**) pup makes *head-over* contact with mother’s back-of-head and *ffl-over* contact with her shoulder.

The occurrence of MP play interaction at Donna Nook seemed to increase in late lactation, consistent with increasing levels of pup general activity and MP interactions previously observed on the Isle of May, Scotland [19,26]. Mother–pup interactions in small pools generally included more sustained play and head-over contact than on dry land (Table 1). Observations (pers. obs.) have suggested that this predilection for play in water also occurs at coastal sites such as West Wales (Figure A1). 

The present Donna Nook observations suggest a greater level of mother-pup interaction than recorded in the earlier studies in Scotland [19,26,27]. However, this is undoubtedly due to different methodologies used, i.e., scan sampling of colony behaviour by the earlier studies and observations focused on suckling bouts in the present study. One of the earlier studies considered that mother-pup interactions were mainly related to the pups seeking the nipples [19]. However, the present study found that most of the pups’ unilateral nosing contacts were to the mother’s face, head, neck, and chest regions, and not to her abdomen. These nosing contacts were increased during play, as demonstrated by the previous finding in harbour seal mother-pup interactions [15]. Nosing contacts were therefore an integral part of play rather than a measure of “non-playful social interest”, as suggested for juvenile rats (*Rattus norvegicus*) [34].

In harbour seal pups, nosing contacts are targeted to the nape or side of the neck and muzzle, likely because the aquatic harbour seal pup orients to these body areas of the mother as they travel in the sea; the target body areas appear to be less specific for the more shore-bound grey seal pups. The sebaceous glands on the mother’s skin are thought to convey odours to the pup when it makes nosing contact [15]. An odour secreted by the sebaceous cells of the nape area elicits locomotor play movements by juvenile voles (*Microtus agrestis*) [35], while touching the nape with the nose is a primary target of juvenile rats during play fighting [36]. Thus odour from the face, head, and neck area may be a potent social attractant in diverse mammal species. 

The gentle touch of hairy skin, as occurs with *fll-over*, likely stimulates *C low threshold mechanoreceptors (CLTMs)*, which transmit via unmyelinated afferent nerves with a conduction velocity of <5 cm/s, which has been called the *affective* or *optimal touch velocity* [37,38,39]. These nerves transmit signals to the emotional systems of the frontal lobe [37]. The CLTM fibres innervate relatively short, fine hairs rather than longer coarser hairs [40]. Possibly, the apparent increase in play responsiveness of grey seal pups towards weaning might be partly due to these gentle-touch sensitive hairs becoming exposed as the overlying lanugo coat is moulted. Stroking at a faster rate when the grey seal mother pushes the pup towards her nipples with a rapid scratching motion likely stimulates the myelinated afferent nerves from the skin, transmitting a direct message to the pup [37] to get on with suckling. 

The mother-pup play bouts seemed more likely and lasted longer in water than on dry land, while the play of grey seal subadults and all harbour seals occurred only when the seals were wet in the water or at the water’s edge. Harbour seals notably tend not to engage in affective gentle touch when hauled out and dry. Possibly, the seals’ affective tactile sense is heightened when the skin is wet, owing to the CLTM fibres’ increasing sensitivity when wet, as described for human hairy skin [41]. 

The endpoint of the Donna Nook grey seal mother-pup play appeared to be engaging in body-to-body contact in which *head-over* contact predominated, along with fore-flipper over (*ffl-over*) contact and occasionally “hugging” (Figure 5). This social contact has been called “affective touch” and is believed to reduce stress levels [39]. The *ffl-over* contact would equate to a “gentle” or “pleasant touch”, whereas the *head-over* touch and hugging would equate to “deep pressure”, providing a “subjective pleasant sensation”; both gentle touch and deep pressure activate different brain cortex regions [42]. The close contact play of pups with their mothers would be expected to result in increased plasma oxytocin levels [43], dopamine reinforcement [44], and activation of rewarding endorphin and serotonergic systems [37]. 

Mother-pup play was only observed at Donna Nook in about 25% of pairs where the pup was past the newborn stage. The main outcome of mother–pup play versus non-play measured in this study was the increased level of body nosing by the pup (Figure 4). However, body nosing contacts by the pup occurred in *pre-suckle* bouts even in the absence of play (Figure 4) and is almost always a preliminary behaviour to suckling [45]. Bekoff [46] has noted that in some species, only minimal amounts of social play early in development are necessary to produce socialised individuals; the typical levels of *pre-suckle* body nosing contacts observed even in the absence of play may therefore be sufficient for normal primary socialisation. The play bouts recorded *post-suckle* or *unrelated* to suckling may provide the pup with “extra” tactile and olfactory input and likely have positive effects on its development.

Bateson [47] has suggested that play may produce a more resilient individual both physiologically and behaviourally. The survival of grey seal pups in their first year has been linked to body condition at weaning [48]. Grey seal pups with relatively high oxytocin levels gain mass at a relatively rapid rate [49]. Since the close nosing and body contact during mother-pup play would be expected to elevate oxytocin levels [43], it follows that mother-pup play may contribute to good body condition at weaning and hence to first-year survival. 

Mother-pup play might also contribute to maximising first-year survival by shaping and refining neural changes to different areas in the frontal cortex, facilitating the development not only of enhanced social skills [50] but also general cognitive abilities [51], benefitting seal pups as they learn to navigate the marine environment and forage. Such an effect may occur in brown bear (*Ursus arctos*) cubs: those who played more in their first summer had better first-year survival [52]. 

### 4.2. Weaned Grey Seal Pups at Donna Nook

**Figure A2:**
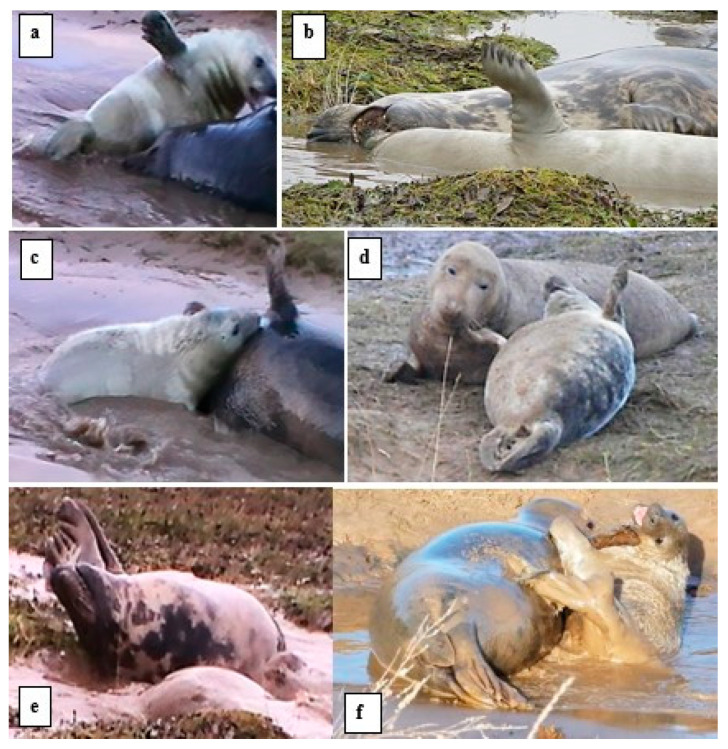
The raised fore-flipper (*ffl*) components of *play-display* during mother–pup play: (**a**) P *ffl* and *open-mouth* “play face”; (**b**) P *ffl*, M *open-mouth*; (**c**) M *ffl*, semi-*supine*, P *head-over*; (**d**) P both fore-flippers raised (*ffl*) while *supine*; (**e**) mother hind-flippers raised (*hfl*) while *supine*; and (**d**) pup hind-flippers and fore-flipper raised (*hfl* and *ffl* together with *open-mouth)*.

**Figure A3:**
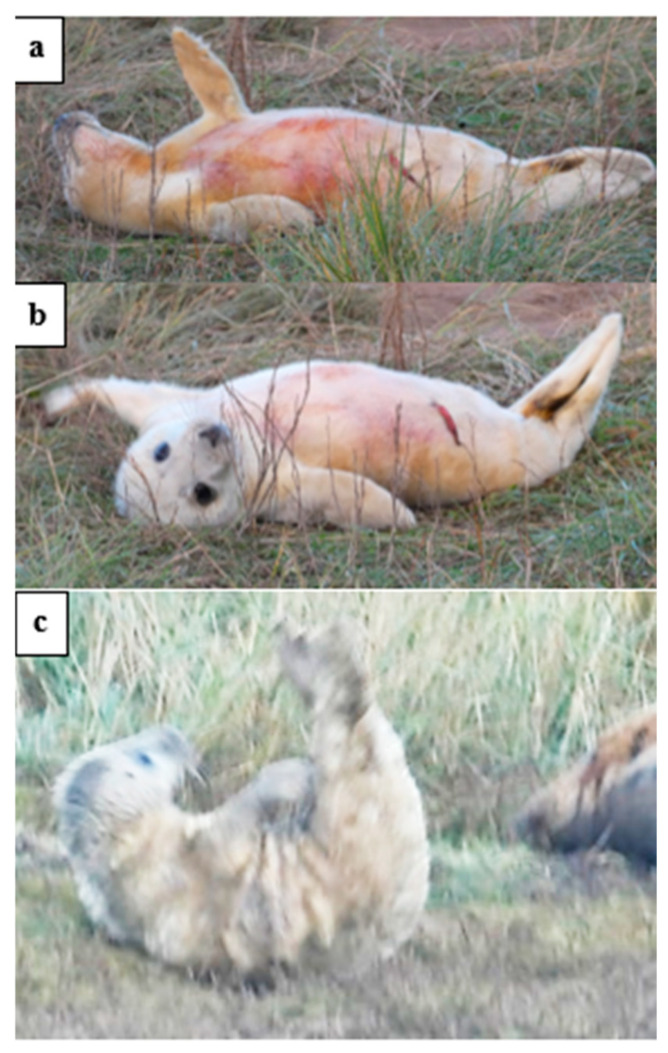
Stretching “exercises” by stage 1 grey seal pups resembling socially orientated postures during *play-display* behaviour of weaned pups: (**a**) lying *supine* with fore-flipper held vertically (*ffl*-up); (**b**) lying *supine*, fore-flippers outstretched and hind-flippers slightly elevated (*hfl*-up); and (**c**) pup raises fore-and hind-quarters simultaneously *(H* + *hfl*).

Following their mothers’ departure for the sea, the pups’ behaviour towards one another could be interpreted as mutually attractive but generally tentative and contact shy. An earlier study [19] concluded that playful movement and splashing were always self-directed, whereas at Donna Nook the *play movements*, *play splashing*, and *play-display* behaviours were mainly in response to the approach or proximity of another pup (Table 4). I suggest that *play-display*, which has not been previously recognised in seals as a social signal, serves to gain attention and communicates an amicable mood, likely inspiring trust in the companions. Play-display components also occurred during mother-pup play (Figure A2). The origins of *play-display* as a social signal possibly evolved through ritualisation from the poses seen in the post-suckling stretching exercises common in stage 1 pups (Figure A3). Such a ritualisation process is manifest in the tail-up signal of amicable intent by domestic cats [53,54]. Moreover, informal observations suggest that stretching by cats when they awake—including in the supine position—may also be a social signal expressed towards humans to communicate affection, relaxation, and sometimes to gain attention (https://www.toolify.ai/ai-news/the-secret-behind-your-cats-stretching-habits-192212; accessed 18 March 2024 https://www.petguide.com/blog/cat/whats-cats-stretching/; accessed 18 March 2024). 

When the mother departs at weaning, the pup’s oxytocin level falls, although it is still similar to its mother’s during lactation [49,55]. Oxytocin levels in weaned pups promote mutual proximity [55,56], as manifested by gatherings of post-weaning pups at Donna Nook. Social recognition between weaned pups following an initial meeting also occurs [29]. The Donna Nook pup group encounters around water pools can therefore provide natural social opportunities for a network of familiar individuals to develop. Initial meetings between individuals involving tentative *whisker* contact and *defensive* splashing would suggest only a defensive rather than a “hostile” or “aggressive” response [28,29]. 

After mutual familiarisation, the Donna Nook weanlings started to follow one another across the salt marsh to the sea and thence started their first individual foraging excursions, which may last a few months [22]. It seems likely that the social interactions among weanlings are not sufficient at this stage to form inter-individual social bonds, which might inhibit individual dispersal [46]. However, during the following spring and summer, grey seal pups assemble at haul-out sites and resume the process of tentative interaction; by six months of age, this develops into play typical of juveniles [28]. 

### 4.3. Subadult Grey Seals

Observations on Ramsey Island (West Wales) suggest an annual or seasonal renewal of acquaintances among subadults is necessary for contact play to proceed. An assembly of juveniles was observed at an entrance to a cave for two weeks from the end of August 1968. Young seals entering or leaving the cave approached each other and made brief nose-to-nose or cheek-to-cheek types of contact, occasionally displaying defensive behaviour but most often separating or following. These seals then moved to a nearby beach, where tentative interactions continued for two days, but from mid-September, dyadic contact play was the prevalent form of interaction [14]. 

It has long been understood that playing animals must negotiate an agreement to play [38,56]. In grey seal subadults, this agreement is negotiated through reciprocation of the *head-over* overture as well as adoption of the *supine* position, fore-flipper over (*ffl-over*) touching, close body contact, and *open-mouth* play face. The pattern of one partner making the *head-over* gesture and the other lying *supine* is reminiscent of the play “pouncing” and “pinning” positions of juvenile rats [51]. In juvenile rats, reciprocity is believed to activate the medial prefrontal cortex in developing social skills [57]. In otariids, consistent reciprocity of play signals is required to prolong dyadic play [5]. 

It seems likely that young grey seals at haul-outs with many others during their subadult years would experience dyadic play with many different partners. Young and adult males outside the breeding season engage in dyadic *rolling* with apparently indiscriminate partners [58]. The experience of multiple play partners and their individual identities is processed in the orbital frontal cortex in rats; such learning is necessary for coordinating sequences of movement with the partner and responses to it [50]. Young grey seals have several juvenile and adolescent years to refine their reciprocity skills in play with many different partners before engaging in sexual behaviour as adults. 

Variation in the developmental refinement of these social skills during play may result in individual variation in mating tactics and success in grey seal males [10,17,59]. Males and females both exercise their social skills in eliciting affective touch during the period of hours or days as a breeding male consorts with an oestrous female [17,32,59]. Mating behaviour often involves the male making *head-over* and *ffl-over* contact with the female (Figure A5). In one observed aquatic mating, a female apparently selected the male and successfully solicited mating using nosing, head, and flipper contact, and the *supine* position [60]. In many non-human mammals, tactile behaviours such as allogrooming occur in association with mating behaviour [37]. It seems likely that this tactile behaviour of grey seals is the phocine equivalent of allogrooming. It therefore, seems likely that refinement of social skills within a network of personal relationships, developed through contact play throughout life, may underscore the social cohesion of the breeding colony, mitigate competition between breeding males, and culminate in affective contact interaction during mating behaviour. 

### 4.4. Grey and Harbour Seals—A Comparative Perspective

Both species—like other phocid seals—have highly precocial neonates and a period of maternal dependency of only a few weeks, which is immediately followed by a nutritionally independent life lasting some 30 or more years. This means that in both species, this short period with the mother must be a critically sensitive period for the pup’s primary socialisation. In both species, the weaned pups and young seals disperse while they learn to forage at sea, but foraging trips in young seals of both species are punctuated by periods assembling onshore where they may engage in intense social play. 

Mother-pup and subadult play in both species was characterised by intense affective touch combined with olfactory contact. Water, or wetness, appears to be obligatory for play or play-like tactile interactions in harbour seals of all ages; in grey seals, water evidently facilitates such interactions but may not be obligatory. This species difference reflects the stronger evolutionary tendency in harbour seals towards a more aquatic mode for all stages of social interaction and reproduction.

A difference in early socialisation of the two species is that although the pups of both species tend to congregate immediately after weaning, grey seal weanlings of this study appeared initially shy and defensive of close contact, whereas harbour seal weanlings appear to transition easily from their relationship with their mother to affiliative social contact with other weanlings [33,61]. This difference may be partly related to separate evolution and demands of ecology since divergence ~2.5 MYA [8], leading to grey seal weanlings’ immediate individual dispersal while they learn to forage, while harbour seal weanlings may forage socially [33,61]. Although the minimal sociality between grey seal pups is suggested (above) to be related to their minimal opportunity for peer contact in their original ice habitat, it would be interesting to study the affiliative behaviours of mother-pup pairs and weanling pups of the largha (spotted) seal, *Phoca largha*, an ice-breeding species that diverged from the harbour seal only ~1.5 MYA [8]. Where the largha seals breed on terrestrial sites, the weaned pups gather in groups, as do grey and harbour seal pups, but some pups apparently associate in pairs over subsequent weeks [62], suggesting more social bonding than in grey seal weanlings. 

**Figure A4:**
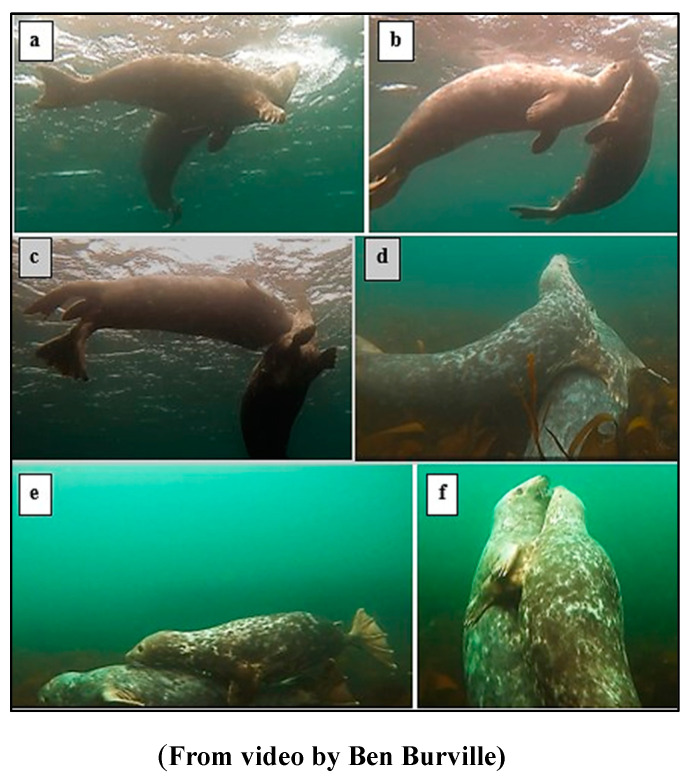
Dyadic contact play underwater by two grey seal subadult males: (**a**) one blows bubbles at the surface, shoulder-to-shoulder contact with the partner; (**b**) mouth-to-mouth contact just below the surface; (**c**) one leans back-of-head against the other’s chin/throat; (**d**) one has fore-flipper(s) over the other, making venter-to-dorsal contact to include chest and shoulders; (**e**) one makes ventral contact from chin to chest and dorsal contact with the partner from shoulder to rump; and (**f**) pair make venter-to-venter contact >50% and one has relaxed *open-mouth*.

**Figure A5:**
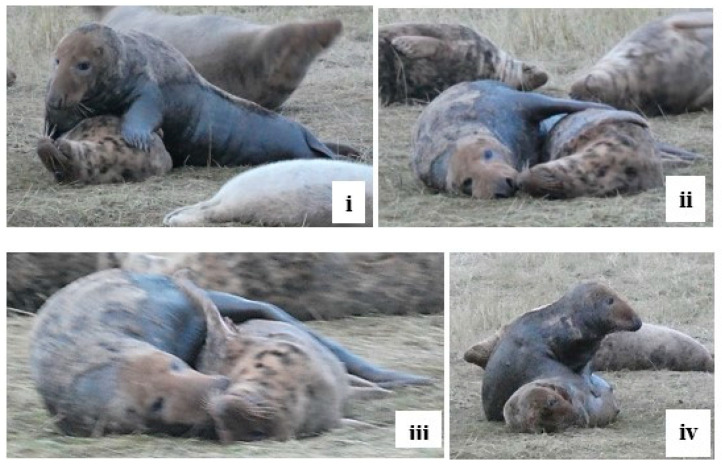
Contact during mating at Donna Nook (2023). Pre-coitus: (**i**) male leans head, shoulder, and makes *ffl-over* contact with female who is *supine* with mouth slightly open; (**ii**) male makes *ffl-over* contact with female, with nose-to-nose and body contact. (**iii**) Coitus: male makes *ffl-over* contact with female; female is *supine* and makes reciprocal *ffl-over* contact with male; and male nuzzles female’s throat. (**iv**) Post-coitus: male leans over female and makes *ffl-over* contact.

**Figure A6:**
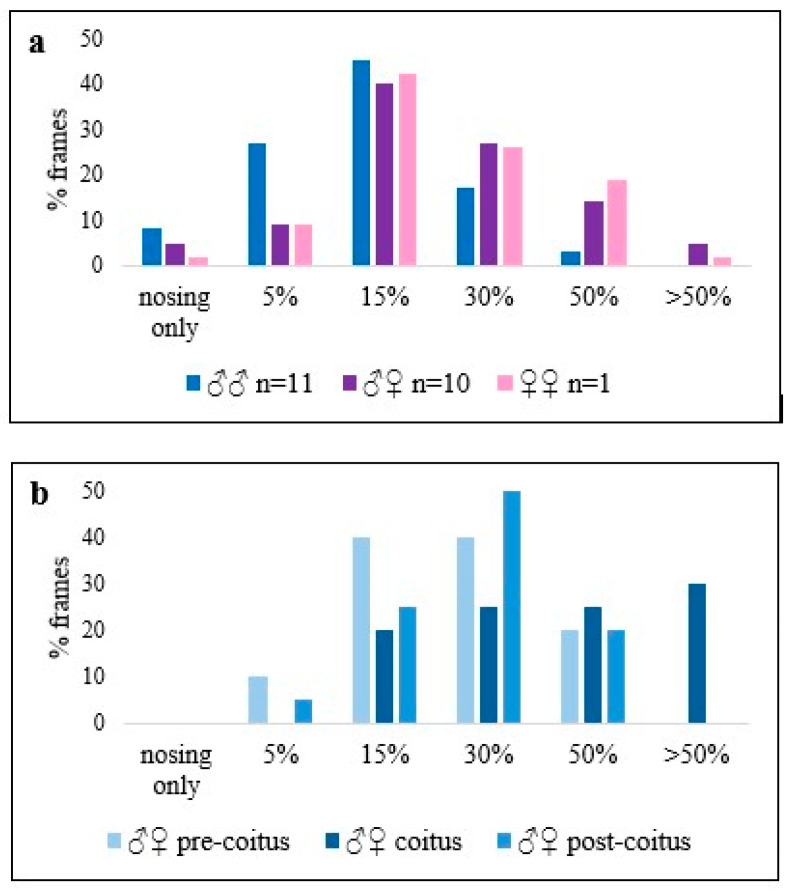
(**a**) The estimated amount of overlapping body contact during (**a**) grey seal subadult play interactions (Red Wilderness and Cemaes Head, West Wales) and (**b**) an adult mating sequence (Pwllderi, West Wales).

It is generally considered that animals play when they are healthy and well-fed [46]. There has been an apparent dearth of aquatic play by juvenile harbour seals in north-east Ireland in recent decades, possibly due to a change to a poorer diet due to a collapse in local herring abundance [63]. However, grey seal play is less physically energetic [13,14] (Figure A4) and therefore requires less energy expenditure; grey seals may also be less vulnerable than harbour seals to changes in local prey availability since they travel further on foraging trips [64]. 

The affective contact behaviour patterns between mother and pup and between subadults are essentially the same in both species, having persisted through the 2.5 million years since their phylogenetic divergence [8]. 

## 5. Conclusions

Close olfactory contact and play-like affective touch in grey seals is a theme beginning in infancy with the mother and continuing intermittently through the years of immaturity to courtship, mating, and—in females—to raising the next generation of pups.

Grey seal mother-pup pairs in this study generally engaged in body-nosing contacts, while some pairs also engaged in contact play. The first break in the developmental continuum of close social contact came immediately after weaning when pups did not immediately transfer their close contact with their mothers to their peers; instead, they went through a period of exploring a new environment and making initial acquaintances with other pups. The defensive behaviours initially occurring in weaned pup encounters suggest this process is stressful. The play-like swimming, splashing, and play-displaying may serve to reduce stress, both in the performer and the spectator, and thereby facilitate preliminary positive social contact.

The consequences of the greater degree of sexual dimorphism and polygyny in grey seals are understood here to be superimposed seasonally on an essentially cohesive social structure, which is only superficially different from that in harbour seals. The commonality of contact play patterns between grey and harbour seals suggests that similar play patterns would also be predicted in other northern seals in the closely related genera *Pusa* and *Phoca*, which have radiated during the past 2.5 million years from a common ancestor. 

## Figures and Tables

**Table 1:** No. of behaviour sequences and play frequency of each sequence type in relation to suckling. Play was defined as at least one *head-over* contact during the interaction bout.

Pup Stage	*Pre-suckle*		*Inter-suckle*		*Post-suckle*		*Unrelated*		Play Freq.
	Total No.	*Play*	Total No.	*Play*	Total No.	*Play*	Total no.	*Play*	
**1nb**	3	*0*	2	*0*	3	*0*	3	*0*	**0.00**
**1**	15	*0*	13	*1*	24	*0*	2	*0*	**0.02**
**2**	5	*2*	10	*3*	21	*4*	5	*1*	**0.25**
**3**	10	*4*	15	*4*	29	*7*	7	*4*	**0.31**
**4/5**	3	*2*	5	*4*	9	*3*	4	*4*	**0.62**
**Play freq.**	**0.22**		**0.27**		**0.16**		**0.43**		

**Table 2:** Nosing scoresd for nose-to-body contacts by the pup to each of the mother’s body regions 1–10 (Figure 5) recorded during play bouts.

	Mother Body Regions									
	1	2	3	4	5	6	7	8	9	10
*Pre-suckle*	15	9	9	2	8	2	4	2	0	2
*Inter-suckle*	51	20	8	20	23	6	11	5	2	21
*Post-suckle*	54	35	18	58	62	5	30	10	4	9
*Unrelated*	38	23	23	16	23	8	11	9	2	3
***Total***	***158***	***87***	***58***	***96***	***116***	***21***	***56***	***26***	***8***	***35***
**Total %**	**23.9**	**13.2**	**8.8**	**14.5**	**17.5**	**3.2**	**8.5**	**3.9**	**1.2**	**5.2**

**Table 3:** Behaviours of weaned grey seal pups at Donna Nook, 2022–2023. Behaviours are listed in order of prevalence (% of occurrence in a total of 449 30 s time segments). Behaviours considered to be specific to play are in bold type.

Behaviour	Rank (%)	Description
*Approach*	1 (32.5)	One P approaches 2nd P, who is not moving
*Follow*	2 (26.3)	One P follows another P who is moving away
*Look at*	3 (25.6)	One P looks at another P
***Play splash***	4 (23.8)	P splashes at the surface using fore- or hind-flippers or the whole body
***Play movement***	5 (16.7)	Exaggerated movements of the head or body, lateral movement, makes waves while swimming, streaks along just under the surface
*Nose-to-nose*	6 (15.4)	2 Ps make nose-to-nose contact, including nose-to-mouth or mouth-to-mouth
***Play display***	7 (14.5)	P raises fore- and/or hind-flipper high in air, and/or stretches head high up or backwards, relaxed body tone
***Supine***	8 (13.4)	P lies on its back, relaxed body tone
*Nose-to-body*	9 (10.2	One P noses the body (anywhere other than the nose) of another P
*Defensive*	10 (8.5)	One P repels another by flippering, splashing at it, snorting, or exhaling audibly
*Whiskers*	11 (8.0)	2 Ps come nose-to-nose with only whiskers touching
***Open-mouth***	12 (7.6)	P opens its mouth slightly or widely
*Nose-to-hind-flippers*	13 (3.6)	One P noses hind-flippers of another P
***Head-over***	14 (1.34)	One P leans its chin or head over or against another P

**Table 4:** Co-occurrence in 30 s time segments of *approach*, *follow*, and *play-display* with six most frequently observed behaviours of weaned pups.

	*Approach*	*Follow*	*Play-Display*
**No. of 30 s segments**	**145**	**117**	**66**
*Look at*	40	38	22
*Play splash*	47	24	34
*Play movement*	39	18	16
*Nose-to-nose*	33	20	10
*Supine*	21	0	35
*Nose-to-body*	18	14	4

**Table 5:** Total observed occurrence of four key play behaviours by grey seal mother-pup (MP) pairs and subadult (SA) ♂♀ dyads.

Grey Seal		*head-over*	*ffl-over*	*supine*	*open-mouth*
MP pairs (Donna Nook)	M	97	38	13	27
	P	65	10	16	33
SA ♂♀ dyads (Red Wilderness)	♂	96	72	6	9
	♀	71	44	24	13

**Table 6:** Comparison between the grey and harbour seal records of the occurrence of four key play behaviours of mother-pup (MP) pairs and subadult (SA) dyads during all play bouts recorded.

**(a) MP Pairs**	***head-Over***	***ffl-Over***	***supine***	***open mouth***
Grey seal (Donna Nook)	97	38	13	27
Harbour seal (Sable Island)	63	37	44	6
**(b) SA dyads (♂♀, ♂♂, or ♀♀)**	***head-over***	***ffl-over***	***supine***	***open mouth***
Grey seal (Red Wilderness)	167	116	30	22
Harbour seal (Sable Island)	98	43	49	18

## Data Availability

Data supporting the reported results may be found in the Supplementary Materials, Tables S1–S9.

## Supplementary Materials

The following supporting information can be downloaded at: https://www.mdpi.com/article/10.3390/ani14142086/s1, Table S1: Hg-DN-MP catalogue.xlsx, Table S2: Hg-DN-MP behaviour.xlsx, Table S3: Hg-DN-MP body nosing scores.xlsx, Table S4: Hg-DN-MP play vs. non-play and nosing contacts.xlsx, Table S5: Hg-DN-WP behaviour.xlsx, Table S6: Abbreviations in Excel data sheets.xlsx, Table S7: Hg play reciprocity subadult and MP.xlsx, Table S8: Pv-SI-MP play behaviour.xlsx, Table S9: Pv-SI-Subadult play behaviour.xlsx.

## Appendix A

**Table A1:** No. of photo frames with each play component in eight grey seal subadult ♂♀ play bouts (Red Wilderness, West Wales, March–April 1986–1987).

Bout Reference	9	15	17	18	19	20	21	23	
No. of Photo Frames	33	20	52	35	20	36	53	22	Totals
♂ *head-over*	14	10	25	18	1	9	16	4	97
♀ *head-over*	6	4	3	7	13	3	23	9	74
No. of swap *h-over*	4	7	5	6	1	11	14	6	54
♂ *ffl-over*	13	2	20	15	1	7	10	2	70
♀ *ffl over*	11	0	7	2	0	9	11	7	47
♂ *supine*	0	0	0	1	3	2	6	4	16
♀ *supine*	2	2	11	13	1	2	1	1	34
♂ n-body	2	1	7	0	1	1	0	0	12
♀ n-body	3	1	3	1	2	3	2	1	16
nose-to-nose	0	0	3	0	0	1	1	0	5
lie venter-to-venter	3	1	2	3	1	2	5	3	20
